# Supplementary material for: Differences in the Course of Depression and Anxiety after COVID-19 Infection between Recovered Patients with and without a Psychiatric History: A Cross-Sectional Study
Source: Int J Environ Res Public Health. 2022 Sep 8;19(18):11316. doi: 10.3390/ijerph191811316 (PMC9517442; doi:10.3390/ijerph191811316)
Supplement: Supplementary file 1 [file ijerph-19-11316-s001.zip › ijerph-1882605-supplementary.pdf]

Table S1. Results of ANCOVA for Depression and Anxiety after Adjustment for Sequelae.

	**Depression (PHQ-9)**						**Anxiety (GAD-7)**					
	SS	***df***	***MS***	***F***	η^2	***P***	***SS***	***df***	***MS***	***F***	η^2	***P***
Psychiatric history	15,131.69	1.00	15,131.69	586.10	0.089	<0.001	10,588.24	1.00	10,588.24	626.32	0.095	<0.001
Time since COVID-19 infection	452.40	3.00	150.80	5.84	0.003	0.001	985.80	3.00	328.60	19.44	0.010	<0.001
Psychiatric history × Time since COVID-19 infection	220.60	3.00	73.53	2.85	0.001	0.036	239.64	3.00	79.88	4.73	0.002	0.003

PHQ-9, Patient Health Questionnaire-9; GAD-7, General Anxiety Disorder-7.
